# Supplementary material for: Identification of the Potential Role of the Rumen Microbiome in Milk Protein and Fat Synthesis in Dairy Cows Using Metagenomic Sequencing
Source: Animals (Basel). 2021 Apr 26;11(5):1247. doi: 10.3390/ani11051247 (PMC8146572; doi:10.3390/ani11051247)
Supplement: Supplementary file 1 [file animals-11-01247-s001.zip › Supplementary Files/Supplementary Figure.docx]

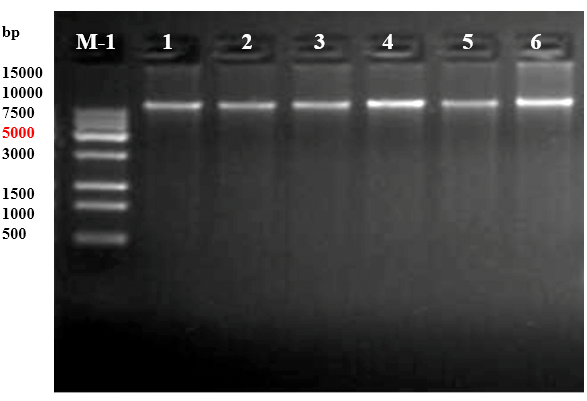


**Supplementary Figure S1.** The 1% agarose el image of 6 samples.

Note: M-1 is 15k marker, 1-6 are high1, high2, high3, low1, low2, low3 rumen fluid DNA sample.


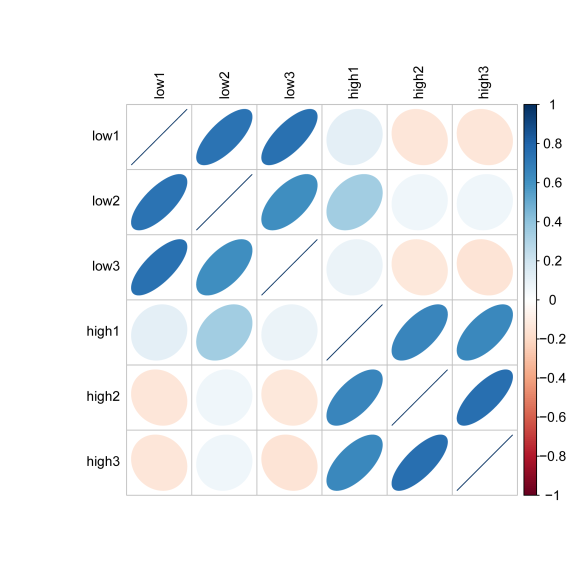


**Supplementary Figure S2.** Heat map of correlation coefficient between samples

Note: In the figure, different colors represent the level of the Spearman correlation coefficient; see the legend on the right for the relationship between the correlation coefficient and the color; the darker the color, the greater the absolute value of the correlation coefficient between samples; the leftward deviation of the ellipse indicates that the correlation coefficient is positive. The bias is negative; the flatter the ellipse, the greater the absolute value of the correlation coefficient.


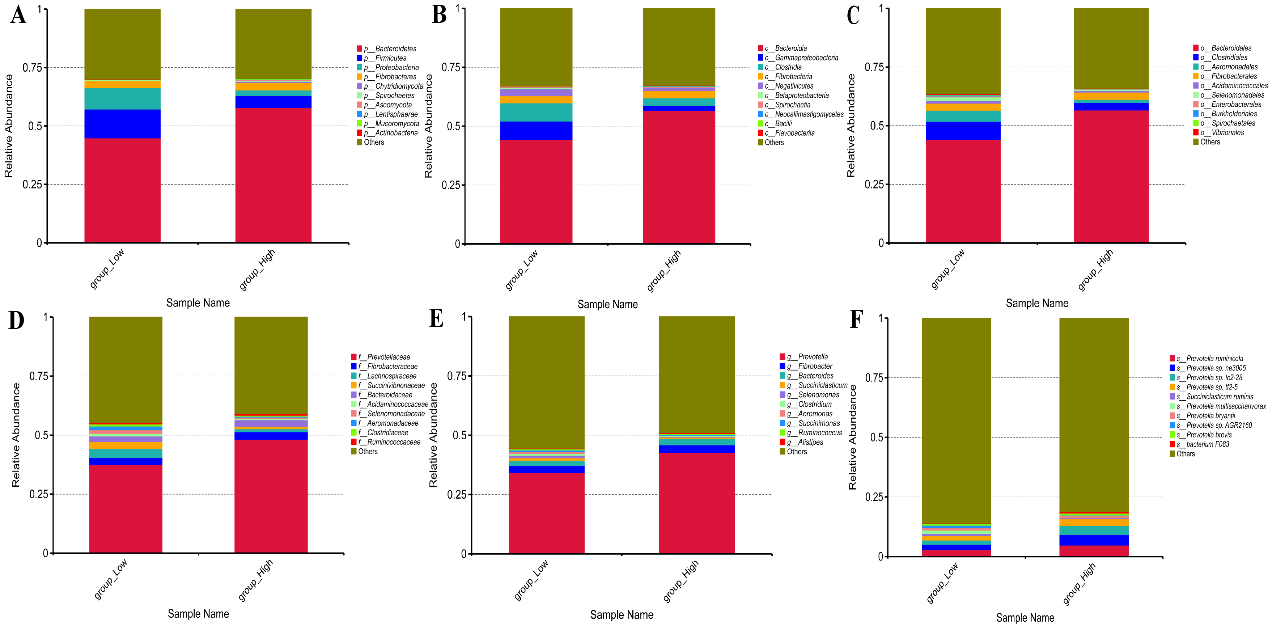
**Supplementary Figure S3.** Composition of the most predominant bacterial in the rumen at different levels.

Note: A to F are phylum, class, order, family, genus and species levels

**Supplementary Figure S4.** Rank abundance curve graph
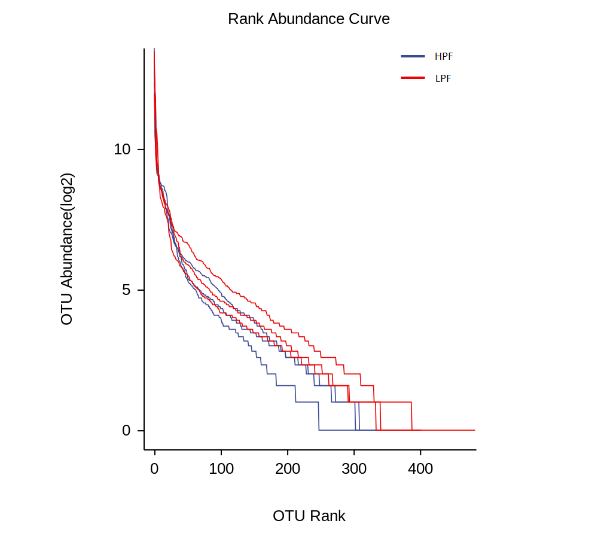


Note: The ordinate represents the abundance of each species in the corresponding sample. Each broken line represents the abundance distribution of a sample at the species level. The length of the broken line on the horizontal axis reflects the number of species in the sample. The longer the broken line, the more species in the sample.
